# Supplementary material for: High-harmonic generation by field enhanced femtosecond pulses in metal-sapphire nanostructure
Source: Nat Commun. 2016 Oct 10;7:13105. doi: 10.1038/ncomms13105 (PMC5062548; doi:10.1038/ncomms13105)
Supplement: Supplementary Information — Supplementary Figures 1-2 and Supplementary Notes 1-2. [file ncomms13105-s1.pdf]

Supplementary Information

High harmonic generation by field enhanced femtosecond pulses in metal-sapphire nanostructure

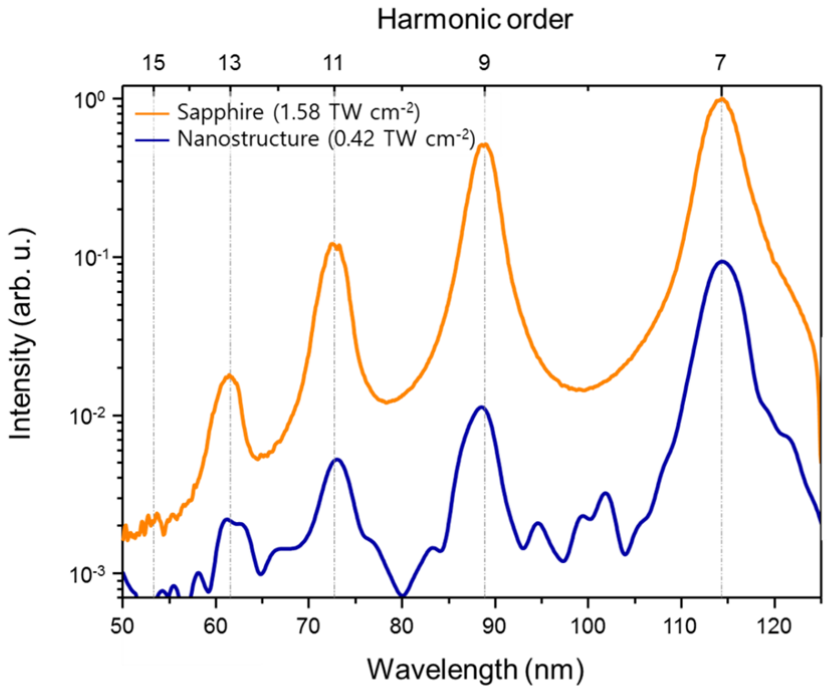

Supplementary Figure 1 | Comparison of EUV spectra between a bare C-plane sapphire and the metal-sapphire nanostructure. The two spectra measured from different targets show a large offset in the EUV yield but demonstrate a strong resemblance.

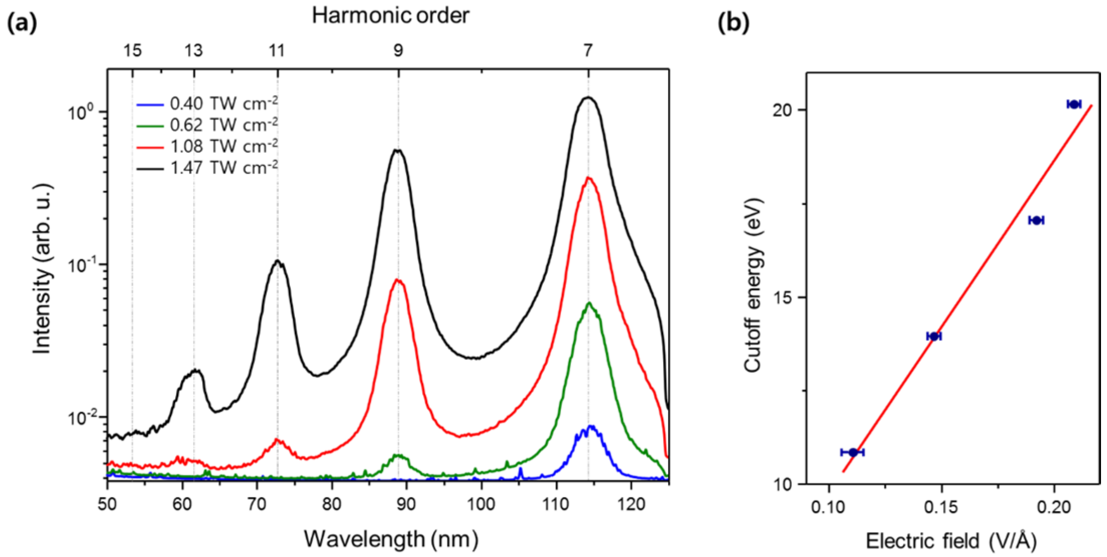

Supplementary Figure 2 | HHG generation and energy cutoff estimation in bare C-plane sapphire. (**a**) Measured EUV spectra with increasing the field intensity. (**b**) Cutoff energy vs. electric field of the driving laser. The cutoff energy for each electric field is given by a blue dot with error bar. The red line is the linear least-square fitting of measured data.

Supplementary Note 1

Supplementary Figure 1 shows a comparison of EUV spectra; one is from the metal-sapphire nanostructure with a driving intensity of 0.42 TW cm^{-2} as described in the main manuscript, and the other is from the bare sapphire with a stronger intensity of 1.58 TW cm^{-2} as reproduced from Supplementary Fig. 2. Both the EUV spectra exhibit harmonics up to H13. When the bare sapphire was illuminated by the same lower intensity given to the nanostructure, only weak harmonics of H7 and H9 were observed as in Supplementary Fig. 2. The EUV yield from the bare sapphire was about two orders of magnitude higher, which was attributed by the large emitting area of a 5 µm diameter; the emitting area of the nanostructure is restricted to a 0.4 µm diameter due to its nanostructure sapphire tip size. Despite the difference in the EUV yield, the two spectra shown in Supplementary Fig. 1 clearly demonstrate a strong resemblance; evidencing the HHG peaks from the nanostructure are not significantly influenced by fluorescence emission from either sapphire or gold. The peak seen between H7 and H9 in the nanostructure's spectrum is reckoned as an even-order harmonic of H8 generated by the steep spatial variation of the enhanced field or due to unknown noise. Direct comparison of the driving intensities of the two spectra indicates that the nanostructure provides an average intensity enhancement factor of 3.8. It is reckoned that the strong field enhancement at the edges of the nanostructure tip offers limited contributions to the measured spectrum because it is narrowly localized and resulting EUV radiation is subject to severe diffractive scattering beyond the acceptance angle of the spectrometer system used in our experiment.

Supplementary Note 2

Supplementary Figure 2(a) shows the HHG spectra monitored directly from a bare sapphire specimen that has no metallic nanostructure for field enhancement at all. The specimen was made of single crystal C-plane sapphire in the shape of a thin plate with a 430 um thickness. The driving laser was focused directly on a 5 µm diameter spot over the specimen with increasing the average power to 280 mW. The polarization direction of the driving laser was aligned to be parallel to the C-place and normal to the A-plane of the sapphire specimen. High harmonics up to the 13th order were clearly observed when the focused intensity reaches 1.47 TW cm^{-2}. Supplementary Figure 2(b) presents the cutoff energy (eV) level of the HHG signals, which linearly scales with the input electric field as noted in the previous HHG works on solids. The cutoff energy was determined as the maximum harmonic peak discernible in each EUV spectrum of Supplementary Fig. 2(a).
